# “One code to find them all”: a perl tool to conveniently parse RepeatMasker output files

**DOI:** 10.1186/1759-8753-5-13

**Published:** 2014-05-01

**Authors:** Marc Bailly-Bechet, Annabelle Haudry, Emmanuelle Lerat

**Affiliations:** 1 Laboratoire Biométrie et Biologie Evolutive, Universite Claude Bernard Lyon 1, CNRS, UMR 5558, Bâtiment Gregor Mendel, 43 Boulevard du 11 Novembre 1918, 69622 Villeurbanne Cedex, France; 2 Atelier de Bioinformatique, Université Pierre et Marie Curie-Paris VI, 4 Place Jussieu, 75005 Paris, France

**Keywords:** Transposable elements, RepeatMasker, Annotation

## Abstract

**Background:**

Of the different bioinformatic methods used to recover transposable elements (TEs) in genome sequences, one of the most commonly used procedures is the homology-based method proposed by the RepeatMasker program. RepeatMasker generates several output files, including the *.out* file, which provides annotations for all detected repeats in a query sequence. However, a remaining challenge consists of identifying the different copies of TEs that correspond to the identified hits. This step is essential for any evolutionary/comparative analysis of the different copies within a family. Different possibilities can lead to multiple hits corresponding to a unique copy of an element, such as the presence of large deletions/insertions or undetermined bases, and distinct consensus corresponding to a single full-length sequence (like for long terminal repeat (LTR)-retrotransposons). These possibilities must be taken into account to determine the exact number of TE copies.

**Results:**

We have developed a perl tool that parses the RepeatMasker *.out* file to better determine the number and positions of TE copies in the query sequence, in addition to computing quantitative information for the different families. To determine the accuracy of the program, we tested it on several RepeatMasker *.out* files corresponding to two organisms (*Drosophila melanogaster* and *Homo sapiens*) for which the TE content has already been largely described and which present great differences in genome size, TE content, and TE families.

**Conclusions:**

Our tool provides access to detailed information concerning the TE content in a genome at the family level from the *.out* file of RepeatMasker. This information includes the exact position and orientation of each copy, its proportion in the query sequence, and its quality compared to the reference element. In addition, our tool allows a user to directly retrieve the sequence of each copy and obtain the same detailed information at the family level when a local library with incomplete TE class/subclass information was used with RepeatMasker. We hope that this tool will be helpful for people working on the distribution and evolution of TEs within genomes.

## Background

Large proportions of eukaryotic genomes are essentially composed of repeated sequences, including the human (approximately 45 to 78% [1,2]), maize (approximately 80% [3]), and salamander (approximately 50% [4]) genomes. Among these repeated sequences, transposable elements (TEs) represent the most significant contributors in terms of sequence coverage and therefore have a major influence on genome evolution, especially on genome size [5]. In contrast to other repeated sequences, TEs consist of a wide diversity of sequences; in addition to the separation in classes based on the transposition intermediate (RNA versus DNA), many subfamilies are described inside each class, corresponding to elements with particular sequence features, and many efforts were made to unify the classification system for all of these elements [6,7].

With the ever-growing number of whole genome sequencing projects, the identification of TEs becomes necessary to fully characterize the evolutionary dynamics of genomes. Different methods of TE identification have been developed during the past 15 years, with the majority designed to determine TE content in assembled genome sequences produced by the classic Sanger sequencing method (for reviews, see Bergman and Quesneville [8], Saha *et al.*[9], and Lerat [10]). These methods group three main types of approaches to recover TE sequences: homology-based approaches that search for a reference sequence in a query genome; structure-based approaches that search for particular structural features of certain TE classes, such as the presence of two long terminal repeats (LTRs) at the extremities of LTR-retrotransposons; and *de novo* approaches that principally use the repetitive nature of TEs to discover them.

More recently, with the emergence of next generation sequencing (NGS) technologies, new efforts were made to develop novel tools to detect TEs because previous methods are not directly applicable to reads produced by NGS data [11,12]. However, one of the most commonly used procedures to find occurrences of known TEs remains the homology-based method proposed by the RepeatMasker program [13] because it is easy to use, rapid, and efficient [14,15]. The main drawback of this program is its dependence on reference sequences and consequent inability to discover new TEs. This method however remains a must for identifying TE sequences in an assembly or after the identification of new consensus TE sequences using *de novo* methods. For example, this last approach (*de novo* TE libraries used with RepeatMasker) was applied for the identification of TEs in the 12 *Drosophila* genomes [16].

The principle of RepeatMasker is to search for the occurrence of any reference sequence contained in a library (currently Dfam [17] and RepBase [18], or user-built in) in a query sequence using a sequence comparison approach based on popular search engines including nhmmer, cross_match, ABBlast/WUBlast, RMBlast, and Decypher [19]. RepeatMasker generates several output files, including the *.out* file, which provides a detailed annotation of all detected repeats in the query sequence, specifically including their position, orientation, and divergence from the reference sequence [19]. This *.out* file is particularly useful because it identifies the part of the query sequence that matches a given TE family of a library (a ‘hit’) and provides its position in the query sequence for each one. However, a remaining challenge consists of identifying the different copies of elements corresponding to those ‘hits’ , which is a prerequisite for any evolutionary or comparative analysis of different copies of a family.

**Figure 1:**
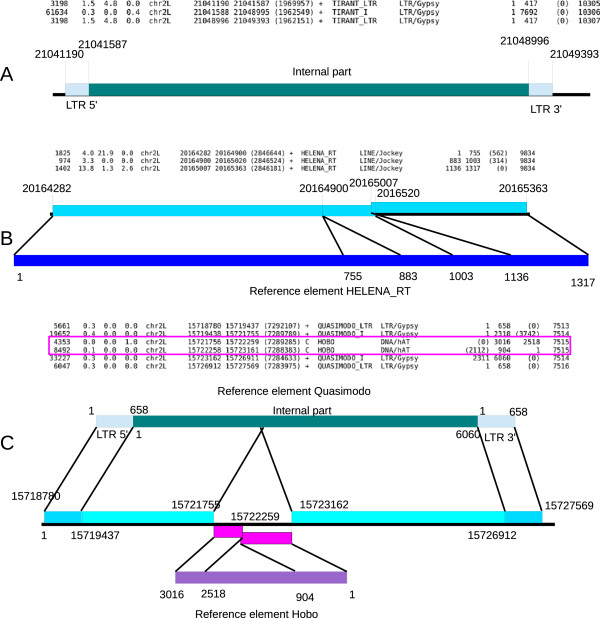
**Examples of multiple hits in the RepeatMasker .*out *file corresponding to one copy. (A)** Multiple hits due to separation between the LTR and internal regions in the consensus sequence in the Repeat Library for the LTR-retrotransposon *Tirant* in *D. melanogaster*. Three hits are obtained with RepeatMasker, which correspond to one complete copy. **(B)** Two large deletions lead to the identification of three hits in RepeatMasker corresponding to one incomplete copy of the *Helena* element in *D. melanogaster*. **(C)** Example of one TE inserted into another. The *Quasimodo* LTR-retrotransposon is inserted in the reverse orientation by a *hobo* element, which is incomplete due to an internal deletion. While six hits are proposed by RepeatMasker, they in fact correspond to two copies, one for each element. LTR, long terminal repeat; TE, transposable element.

Some scenarios in particular can lead to multiple hits corresponding to a unique copy of an element. The first scenario, in the case of a LTR-retrotransposon, comes from the split of its consensus into a sequence corresponding to the LTR and a sequence corresponding to the internal portion of the element (Figure 1A). This separate annotation for LTR-retrotransposons is supported to facilitate the identification of solo-LTRs, which may be numerous in some genomes [20]. Multiple hits corresponding to only one copy of a given element can also result from large deletions (Figure 1B) or insertions that occur in sequences and disrupt the entire copy, leading to nested TEs (Figure 1C). Moreover, the presence of undetermined bases, which may occur due to low sequencing quality, could also disrupt unique sequences corresponding to a copy and give multiple hits. Taken together, these characteristics induce multiple hits corresponding to a unique copy for a given TE in the RepeatMasker *.out* file. Finally, non-significant hits can be present in the output file, in addition to sequences that do not fit the 80-80-80 rule [6], that is, sequences that would align with the reference on less than 80 bp, on less than 80% of their respective length, and with less than 80% of identity.

Some programs proposing the use of RepeatMasker output files were developed [21-23], but none allow access to the location of all of the TE copies or an accurate quantification of the family content at the genomic level. These programs usually have very specific aims. TSDFinder was developed to refine the coordinates of long interspersed nuclear element (LINE) L1 insertions by identifying flanking target site duplication (TSD) sequences and the poly(A) tails of 3′ intact L1 insertions in the human genome [21]. The LTR-miner program was designed to specifically retrieve information concerning the age and distribution of LTR-retrotransposons [22]. This program was then implemented in the Reannotate program for use on all categories of TEs to estimate the temporal order of insertions in the case of nested elements and to estimate the age of LTR-retrotransposon copies [23].

In this manuscript, we propose a perl tool (available at http://doua.prabi.fr/software/one-code-to-find-them-all) that parses the RepeatMasker *.out* files to accurately determine the number of TE copies found, obtain their positions, and retrieve their sequences. This tool should be helpful for any non-bioinformatics scientist interested in genome annotation and/or evolution. To our knowledge, this program is the first multi-purpose tool that correctly identifies TE copies using RepeatMasker and provides complementary quantitative information for individual families in a query sequence.

## Implementation

The proposed tool consists of two perl scripts that must be run successively to take into account the different characteristics of the consensus sequences.

### Script 1: *build_dictionary.pl*

This script builds a list of all of the LTR-retrotransposons found in the query sequence at least once by RepeatMasker to associate hits corresponding to the internal portion and those corresponding to LTR sequences. This module uses the RepeatMasker *.out* file or a directory path containing several RepeatMasker *.out* files as input. RepeatMasker files are recognized based on their *.out* extension, allowing the program to run recursively on large file structures without prior file sorting (for example, working on one organism by running the program on an entire directory downloaded from a genome database). Then, the program matches together internal and LTR portions, based on name similarity. The main issue with this step relies on heterogeneity in the annotation of LTR-retrotransposons in the library. For example, the majority of LTR-retrotransposons in *Drosophila melanogaster* appear under the name ‘TE_LTR’ and ‘TE_I’ for the LTR sequence and the internal sequence, respectively. However, the members ‘LTR’ and ‘internal portion’ may sometimes have different names. This scenario is, for example, the case for the LTR-retrotransposon *HMS-beagle*, for which the corresponding LTR sequence is labelled *DMLTR5*, while the internal portion is labelled *HMSBEAGLE_I*. The same problem occurs more frequently for retrotransposons in *Homo sapiens*, making it difficult to derive a completely generalized algorithm to determine the concordance between the LTR and internal portions. These issues imply that the output file of *build_dictionary.pl* must be manually inspected to correct for any mis-association.

In the standard version (see --*fuzzy* option for the alternative version), the program only recognizes similar names in addition to the ‘LTR’ or ‘int’ suffix or prefix, taking into account small discrepancies such as a ‘-’ symbol replaced by a ‘_’ symbol, for example, recognizing the association between *HERV-Fc2-int* and *HERV-Fc2_LTR*.

The following parameter must be provided in the program:

*--rm* infile (corresponds to a RepeatMasker *.out* file or the name of a directory containing several RepeatMasker *.out* files).

Three options can be specified by users:

--*help*

This option prints a summary of the different usages of the script.

--*fuzzy*

This option allows the script to associate more LTR names with internal counterparts to account for the possibility of LTR variants. In three successive passes, the program associates similar names differing by a single letter, a single number, or two characters. For example, in the human genome, the --*fuzzy* option allows for the association of MER66-int with its various counterparts MER66A, MER66B, MER66C, and MER66D or HERV1_I-int with HERV1_LTRa, HERV1_LTRb, HERV1_LTRc, HERV1_LTRd, and HERV1_LTRe.

--*unknown*

To be used in particular cases where the RepeatMasker program was run using a local TE library without the class/subclass specification (see below).

Finally, the name and path of the output file should be specified using a redirection (>dictionary_output.txt). Examples of command lines are detailed in the tutorial available on the program website.

### Script 2: *one_code_to_find_them_all.pl*

The second script uses the output file produced by *build_dictionary.pl* and a RepeatMasker *.out* file (or a directory containing several RepeatMasker *.out* files). The principle of this program is to compare the positions and orientation of each hit corresponding to the same TE family to determine whether the hits correspond to the same copy and can be merged or correspond to different copies. Two hits located on the same scaffold or chromosome are considered to be fragments of the same copy if they abide by the three following conditions: 1) they have the same orientation; 2) the extremities of the fragments respect a distance criterion: by default the furthest extremities should be separated by less than twice the length of the reference TE element (see the *--insert* option for non-default behavior); and 3) the second fragment starts and ends after the first one respectively starts and ends (that is, the two fragments can overlap but cannot be included in one another). These constraint filters were motivated by a conservative choice, meaning not to merge copies that do not belong to the same insertion. However, one shortcoming of this methodology is that it may be impossible to re-assemble old copies in which many insertions of other elements had taken place after this copy was first inserted in the genome. Moreover, we may over-estimate the copy number if a portion of a given copy is inverted, leading to several fragments in different orientations.

The identification of unique copies of LTR-retrotransposons depends on the different fragments and different portions of the element (LTR and internal portions), as follows. First, we identify different fragments of the same portion that could be later assembled as a copy. For that purpose, two LTR fragments must not be separated by a compatible internal fragment, and two internal fragments must not be separated by a compatible LTR fragment. These steps are necessary for the merging of fragments into a copy. Once all copies are reconstructed from the RepeatMasker hits, the program assembles full-length LTR-retrotransposons by associating LTR copies and their corresponding internal copy located closely to one another. Conditions for associating a LTR sequence with an internal sequence include the following: the LTR sequence must be in the same orientation as the internal sequence, and it must be separated from the internal sequence by less than half the LTR length. The reconstruction of full-length ‘LTR-I-LTR’ elements is performed as a priority, and with the remaining copies, incomplete ‘LTR-I’ or ‘I-LTR’ elements are then built. All copies, assembled or solo, are reported. As solo-LTRs are of special evolutionary interest, they are reported separately from the full-length and partial LTR-retrotransposon copies in the summary file *.copynumber.csv* (see below).

The parameters required by the program include the following:

--*rm* infile (corresponds to a RepeatMasker *.out* file or the name of a directory containing several RepeatMasker *.out* files).

--*ltr* output file from *build_dictionary.pl* (Script 1).

Several options can be specified by users:

--help

This option prints the possible usages of the script.

--strict

This option makes the program use a rule based on the 80-80-80 rule [5] to select hits. In this case, the program provides copies with sizes greater than 80 bp long and which have greater than 80% identity to the reference element. By default, the program gives all hits found, regardless of the size or percentage of identity compared with the reference.

--length ‘length_file’

This option allows users to work with their own file for the length of the reference elements, which will be used to determine the ratio of the length of a given copy compared with its reference. If not provided, the code computes the length of all elements (LTR and internal portions separated for the LTR-retrotransposons) present in all *.out* files under study, by selecting for each element the most common consensus length (as in some cases multiple RepeatMasker consensus sequences can correspond to the same element). This option is valuable when working with elements whose annotation is ambiguous to ensure that the correct reference length is used. It can also be used with another purpose, when only a subset of TEs is considered, since only the elements mentioned in the *.length* file will be taken into account.

--choice

**Figure 2:**
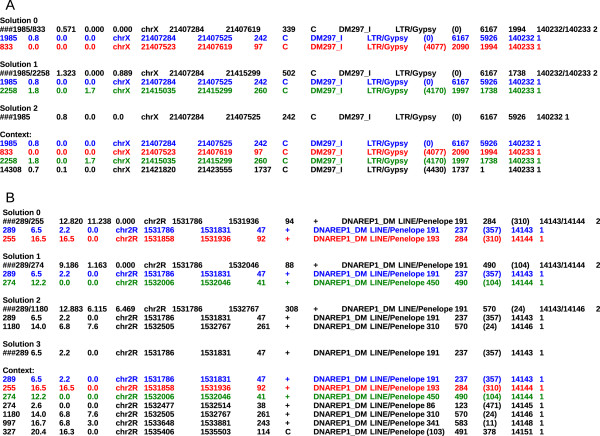
**Two examples of choice as proposed by the program. (A)** The best global solution appears as a concatenation of blue, red, and green fragments. Because the program works locally only on a pair of copies, the best choice to start with is 0, assembling blue and red; after this selection, the program will automatically detect that the assembled blue-red could be concatenated with the green fragment and either propose it to the user if there is ambiguity or assemble them together if the case is unambiguous. **(B)** The best global solution appears to be the concatenation of blue and green (solution 1) rather than blue and red (solution 0).

This option allows users to manually resolve ambiguous situations by choosing their favorite solution for merging hits. For example, Figure 2A shows a case in which two choices are possible, that is, two different hits can be assembled with the one under study (*DM297_I* at position 21,407,284 on the chromosome X). In this case, the first choice (solution 0) is the most parsimonious. Solution 0 is always the one corresponding to assembling closest hits together. However, this solution may come to a fault in the case of multiple nested or duplicated TEs corresponding to the same reference element. For example, in Figure 2B, solution 1 is the most parsimonious, that is, the one that minimizes the reorganization of the copy compared to the reference element structure. If this option is not specified, the default choice consists of choosing solution 0.

As many ambiguous cases can arise, the RepeatMasker block ID (column 14 of RepeatMasker *.out* file) is used when this option is activated. These IDs come from the ProcessRepeats script implemented in RepeatMasker, which makes educated guesses if any pair of fragments is derived from the same element or not. Therefore, if an ambiguous situation can be solved unequivocally using these Block IDs, no choice is left to the user, and the elements sharing the same Block ID are merged.

Another way of quickening the choice process is to only ask the user about ambiguous cases, and sometimes a single choice can disambiguate multiple situations. For example, consider the situation for which three fragments A, B, and C are considered for merging, and for which the choice is between A-B and A-C (choices are always pairwise). If the user considers the right choice to be A-B-C, he/she will choose A-B. Then, if adding C to the merged A-B is not ambiguous (if there is no D fragment of the same element nearby to get confused with, for example), the code will directly merge C with A-B, getting the right result A-B-C without asking the user about this final merging.

--dry-run

This option performs all operations, but reports no results except the log file with all operations performed. It is designed to be used in tests, particularly those determining the number of ambiguous situations to be resolved. Running the program with this option before the actual analysis allows estimation of the time required to complete an analysis with the *--choice* option because the number of ambiguous situations can be high, and manual choice is time-consuming if applied to all elements in a genome.

--unknown

In the particular case in which the RepeatMasker program was run using a local library that did not use the naming system required to differentiate the class and the subclass (required format is described in RepeatMasker help file), the *.out* file is slightly different because column 11 (repeat class/family) is usually filled with ‘Unknown’ or ‘Unspecified’ , which means that the type of individual TE is not specified. To account for this possibility, the user can use the --*unknown* option, which will produce results for elements annotated as ‘Unknown’ or ‘Unspecified’ and deriving from the local, unannotated bank.

*--fasta *and *--flanking ‘size_in_bp’*

The --*fasta* option allows for the retrieving of sequences of copies reported by the program from the local fasta sequence files used in the RepeatMasker program. To study flanking sequences of the determined copies, the --*flanking* option can be specified to allow the program to report the flanking regions of the specified size surrounding each copy in addition to the TE sequence.

--insert ‘size_in_bp’

This option changes the code behavior for merging fragments into copies. By default, the furthest extremities of the considered fragment to be merged are compared, and merging takes place if they are less than twice the reference element length apart. Using *--insert*, the size of the genomic sequence between the two closest extremities of the considered fragments (that is, the size of the insertion between them) will be considered: if it is less or equal to the size given in the option, the fragments are merged. For example, using *--insert* 0 means only fragments detected right next to each other in the query sequence will be considered as parts of the same copy.

By default, five output files are generated, which are located in the same directory as the RepeatMasker *.out* file(s), plus one output file located in the working directory (*.length* file) that is produced only if the *--length* option was not specified.

The *.log.txt* file contains the screen output of the program. For each element, this file summarizes the number of hits and copies obtained after merging the hits. When the --*dry-run* option is chosen, it displays the possible choices that would be asked using the --*choice* option.

**Figure 3:**
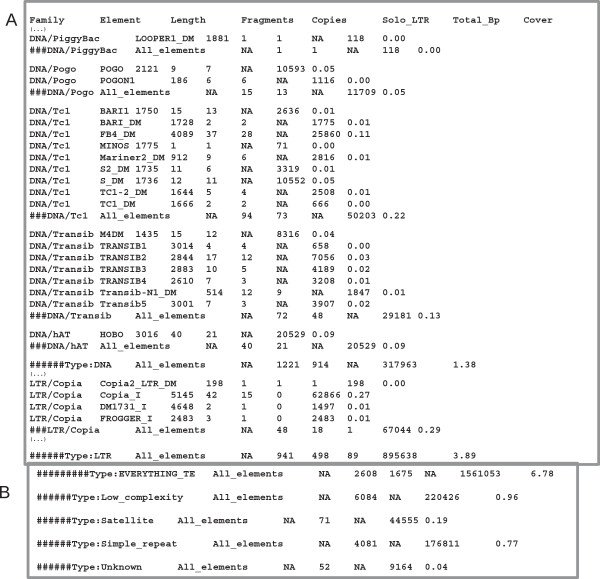
**An example of the output file *.copynumber.csv.*** The tabulated format allows easy reading in many software programs commonly used to plot graphs, such as Excel or R. **(A)** The beginning of the file displays different DNA transposon and LTR-retrotransposon families. Rows starting with ### summarize the information for the above families. **(B)** The end of the file displays a summary for all of the repeat types. LTR, long terminal repeat.

The *.copynumber.csv* file contains quantitative information about each of the identified TE families in the query sequence. This file displays eight columns (see Figure 3A as an example corresponding to some DNA transposons and LTR-retrotransposons detected on the long arm of the chromosome 2 (2L) of *D. melanogaster*): column 1, Family, category of the given TE (as specified in the column 11 ‘repeat class/family’ of the RepeatMasker output file); column 2, Element, name of the given TE (as specified in the column 10 ‘matching repeat’ of the RepeatMasker output file); column 3, Length, length of the reference TE in bp (information from the consensus sequences, as found in the *.length* file). In the absence of either the internal or LTR portion of a LTR-retrotransposon in the query files, the column will specify ‘*No_ref_available*’; column 4, Fragments, number of hits found by RepeatMasker corresponding to a given TE; column 5, Copies, total number of copies reconstructed from the hits (if the --*strict* option was selected, this number can be null, meaning that none of the fragments passed our 80-80 rule); column 6, Solo_LTR, number of solo-LTRs reconstructed from the hits. The column will specify ‘*NA*’ for non-LTR elements; column 7, Total_Bp, total number of base pairs corresponding to a given TE for the analyzed query sequence; and column 8, Cover, percent coverage of a given TE in the analyzed query sequence.

For each TE category (DNA transposons, LINEs, short interspersed nuclear elements (SINEs), and LTR-retrotransposons), the global information concerning the number of fragments, number of copies, number of base pairs, and percent coverage are given and correspond to lines beginning with ‘######Type:DNA’, ‘######Type:LINE’, ‘######Type:SINE’ , and ‘######Type:LTR’. The column ‘length’ in this case contains a *NA*. For example, in Figure 3A, the DNA/hAT transposon *hobo* (reference length of 3,016 bp) has 40 fragments on chromosome 2L corresponding to 21 copies. These copies span 20,529 bp on chromosome 2L, which represents 0.09% of this chromosome. The end of the file gives global information concerning all TEs (and thus the coverage of all TEs on the analyzed sequence), satellites, low complexity regions, simple repeats, and unknown repeat elements (see Figure 3B).

**Figure 4:**
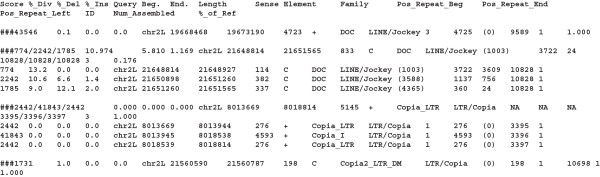
Examples of the output files *.transposons.csv and *.ltr.csv. Lines starting with ### are full copies. Fragments that have been used to assemble the copy are indicated below these lines.

The *.*ltr.csv* and *.transposons.csv files (see Figure 4 as an example) contain the list of all occurrences of LTR-retrotransposons, and of non-LTRretrotransposons and DNA transposons, respectively, which were identified by the program. In these files, the columns globally correspond to those proposed in the RepeatMasker .*out* file, with the exception of the sixth and the last two columns. The (left) column of the RepeatMasker file, the sixth one, is replaced with the length of the reconstructed copy, from the consensus point of view (that is, it can be different from the span on the query sequence). The ‘Num_Assembled’ column corresponds to the number of hits assembled into the different copies. The ‘%_of_Ref’ column represents the proportion of length of the reconstructed copy compared to the reference element. This ratio is expected to be 1 if the reconstructed copy is the same length as the reference element. These numbers thus provide information about the integrity and quality of the copies inserted in the genome; that is, for a given family or superfamily, copies which are mostly full-length (ratio close to 1) and with low divergence from the reference, could result from recent insertion events. In the case of solo-LTRs, that is, copies that only correspond to the LTR section of a consensus, the ratio is computed in reference to the length of the LTR sequence. This implies that full-length solo-LTRs will have a ratio of 1.

Individual copies of TEs correspond to lines beginning with the # character followed by the identification number of the merged hits with each one separated by a slash (/). For those that have been reconstructed using several hits, the fragments used to build the considered copies are shown below. For each copy, the ‘%_Div’ (percentage divergence to the reference), ‘%_Del’ (percentage of deletion compared to the reference), and ‘%_Ins’ (percentage of insertion compared to the reference) are the means of the values of each fragment normalized by size.

In the example in Figure 4, the first copy corresponds to a single fragment of a *Doc* element, which is nearly complete, whereas the copy below has been reconstructed using three fragments that also correspond to a *Doc* element. The third example corresponds to a complete copy of the *copia* LTR-retrotransposon, which has been reconstructed with respect to the separation of the ‘internal portion’ and ‘LTR’ in the consensus library. The last example corresponds to a full-length solo-LTR of *copia2*. The position of each copy is provided in columns 5, 6, and 7, which correspond to the name of the query sequence, position of the start of the copy in the query sequence, and position of the end of the copy in the query sequence, respectively. The orientation on the strand (+ or complementary) is specified in column 9. In the example in Figure 4, the reconstructed copy of the *copia* element is located in the long arm of chromosome 2 (chr2L), starts at position 3,073,087, ends at the position 3,078,231 (is 5,145 bp long), and is on the positive strand (+).

The last output file, *.elem_sorted.csv*, contains the same information as the two previous ones, but sorted per genomic position and not per element, in order to be easily used by people interested in the genomic context and distribution of TEs.

## Results and discussion

To determine the accuracy of the program, we tested it with several RepeatMasker *.out* files corresponding to two organisms, *D. melanogaster* and *H. sapiens*, for which the TE content has already been largely described and which present great differences in genome size, TE content, and TE families.

### Test of the *D. melanogaster* genome RepeatMasker output files

We retrieved the RepeatMasker *.out* files (thereafter mentioned as UCSC files) from the UCSC Genome Bioinformatics website (http://genome.ucsc.edu/), which were produced using version dm3 (April 2006) of the genome sequence with the 17 May 2007 (open-3.1.8) version of RepeatMasker and library release 20061006. Each file corresponds to a different chromosome (2L, 2LHet, 2R, 2RHet, 3L, 3LHet, 3R, 3RHet, 4, U, Uextra, X, XHet, and YHet). We did not retrieve the file corresponding to the mitochondrial genome. We also retrieved the unique RepeatMasker *.out* file (thereafter mentioned as RM file) provided for the same genome version on the RepeatMasker website (http://www.repeatmasker.org) using the library release 20080611 and open-3.2.5 version of RepeatMasker. This file contains the results for all chromosomes.

### Determining the number of ambiguous cases that may require manual inspection (option --*dry-run*/--*choice*)

The option --*dry-run* was used with the UCSC files to determine the number of ambiguous cases that could be manually expertized. For all chromosomes, 862 cases appeared (see Additional file 1: Table S1 for individual chromosome detail). We investigated the cases corresponding to chromosome 3R for which eight ambiguous cases were identified. For all but two cases, the default solution 0 was the best choice from a biological point of view (minimizing the reorganization of the copy compared to the reference element structure). For the two remaining cases, the best choices were solution 1 and the last solution (not assemble the fragments). For chromosome X for which 14 ambiguous cases were indicated, solution 0 was the best choice in ten cases and the last solution (to let the first fragment alone) was the best choice for four cases. This result indicates that the default choice made by the program is the best choice (the most biologically sound) in the majority of cases.

### Running the program with and without the --*strict* option

We did not initially specify use of the --*strict* option and successively ran the program with the UCSC and RM files. When the --*strict* option is not specified, the program considers every hit without filtering using our 80-80 rule. We observed the same amount of TEs globally (both in terms of copy number and chromosome coverage, see Additional file 2: Table S2 and Additional file 3: Table S3) for the two versions of the Repeat Library used with slightly more copies detected in the RM file (208 more copies, see Additional file 2: Table S2). This observation can be explained by the fact that the library used in this case was more recent and thus capable of containing new reference elements. In the results from the UCSC files we observed that the *DNAREP1* element was associated with the repeat class family *LINE/Penelope,* as proposed when it was first described [24], whereas it is now known to correspond to the repeat class family *DNA/Helitron*[25]. In the annotation from the RM file, the association is correct, indicating that the Repeat Library used by UCSC incorrectly assigned this element to the *LINE* category, which was later corrected in a new version. We therefore chose to consider only the output file from the RepeatMasker website (RM file) for the rest of the test. This underlines the importance of a correct TE classification to obtain an accurate amount of particular elements.

Table 1 displays the number of copies per chromosome with and without the use of the --*strict* option. As expected, the global number of copies decreased from 9,134 to 5,656 copies in the euchromatin portion of the genome when the 80-80 rule was applied. This last number is congruent with the 5,409 annotated copies in the *D. melanogaster* euchromatin in the FlyBase annotation version r5.49 (http://flybase.org) [26]. The results also showed that the copy number in unplaced chromosomes is particularly high, indicating that the euchromatin is far from a complete reflection of the entire genome in terms of TE content. While heterochromatin regions display less TE copies (5,066 copies without the --*strict* option and 3,451 copies with the --*strict* option), TEs represent a large coverage of these regions (approximately 60% on average, see Additional file 4: Table S4).

**Table 1:** **Copy number per chromosome for each category of TEs in *D. melanogaster***

**Chromatin type**	**Chromosome**	**Without --*strict *** **option**				**With --*strict *** **option**			
		**DNA**	**Non-LTR**	**LTR**	**Total**	**DNA**	**Non-LTR**	**LTR**	**Total**
Euchromatin	2L	914	263	498	1,675	589	204	278	1,071
	2R	1,018	343	707	2,068	649	246	357	1,252
	3L	892	369	771	2,032	595	289	393	1,277
	3R	549	125	455	1,129	289	91	247	627
	4	626	77	35	738	431	54	21	506
	X	720	187	585	1,492	454	137	332	923
	Total	4,719	1,364	3,051	9,134	3,007	1,021	1,628	5,656
Heterochromatin	2LHet	46	23	88	157	38	21	65	124
	2RHet	462	441	811	1,714	370	372	424	1,166
	3LHet	463	339	708	1,510	366	274	357	997
	3RHet	363	319	683	1,365	282	276	375	933
	XHet	65	54	21	140	43	37	14	94
	YHet	46	45	89	180	37	41	59	137
	Total	1,445	1,221	2,400	5,066	1,136	1,021	1,294	3,451
Unplaced	U	909	1,589	3,365	5,863	777	1,399	2,334	4,510
	Uextra	2,132	8,253	11,827	22,212	1,866	7,668	9,635	19,169
	Total	3,041	9,842	15,192	28,075	2,643	9,067	11,969	23,679
Total		9,205	12,427	20,643	42,275	6,786	11,109	14,891	32,786
Total (without unplaced)		6,164	2,585	5,451	14,200	4,143	2,042	2,922	9,107

LTR, long terminal repeat; TE, transposable element.

Using the output files *.transposons.csv and *.ltr.csv, which contain details for the copies for each heterochromatin chromosome, we retrieved all of the potentially full-length elements by selecting copies whose ratios compared with the reference were over 95% (%_of_Ref, column 17). We obtained 474 copies corresponding to this criterion, which is more than the 202 full-length elements previously described [27] but that includes 130 full-length solo-LTRs. We did the same to determine the number of potentially full-length elements in euchromatin regions and found a total of 655 elements (1,039 elements when counting the highly represented *DNAREP1*, which is no more active and full-length solo-LTRs (170 copies)). This number is higher than the 478 full-length elements described with an older version of the *D. melanogaster* genome, which annotated only 1,572 TE copies [28]. This result demonstrates that our program can quickly identify potentially full-length elements.

In terms of proportion, the global TE content on chromosomes is congruent with what was previously shown [26,27] with an average of 6.69% (6.04% with the --*strict* option) of TEs in euchromatin regions (without taking into account chromosome 4) and 61.63% (52.53% with the --*strict* option) of TEs for heterochromatin regions (see Additional file 4: Table S4).

**Figure 5:**
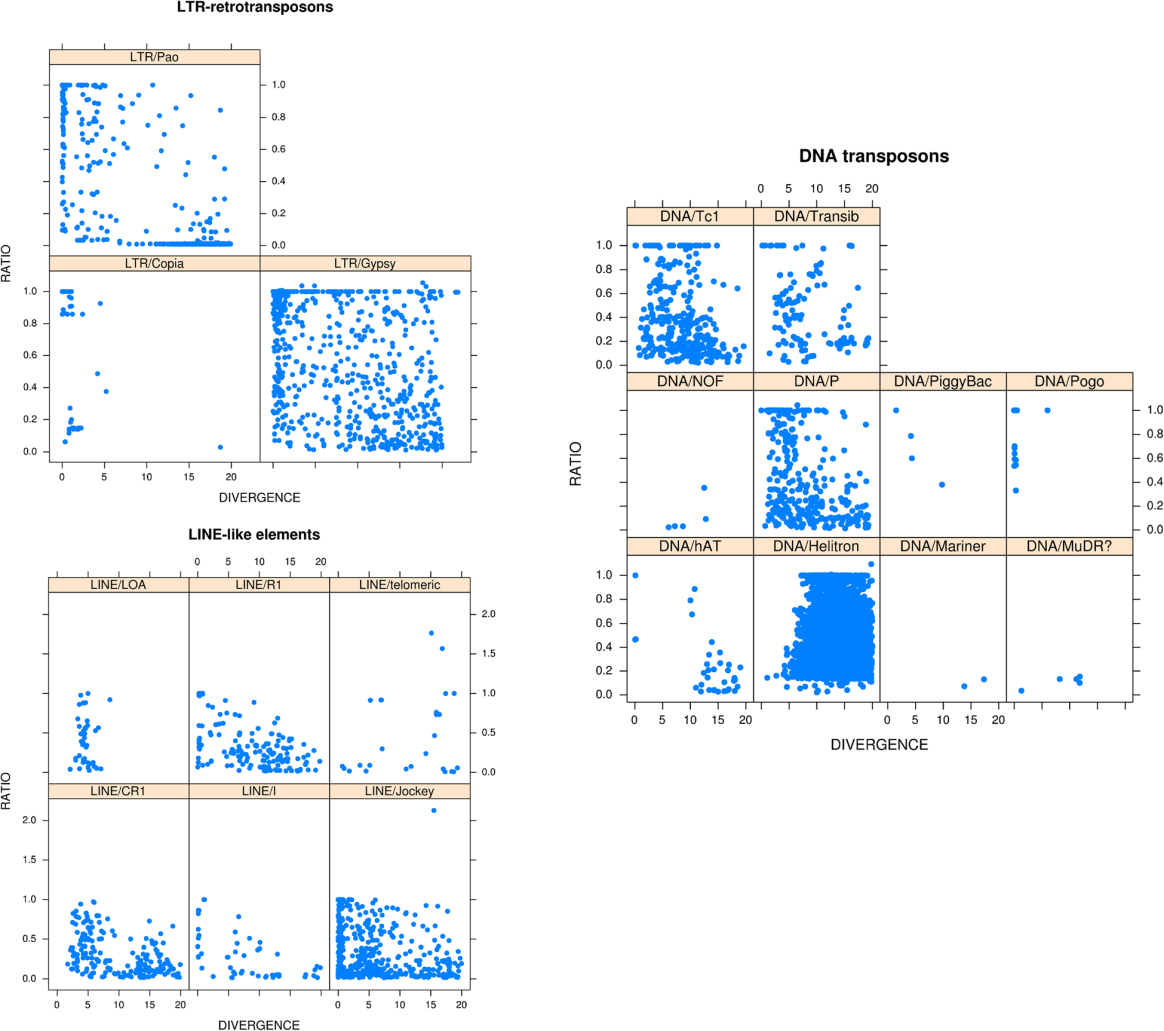
**Plot of the divergences according to the size ratio.** The divergence (column %_Div in files *.transposons.csv and *.ltr.csv) of sequences has been plotted against the size ratio of the copy compared to the reference element (column %_of_Ref in files *.transposons.csv and *.ltr.csv) given for each superfamily of LTR-retrotransposons (upper left panel), non-LTR-retrotransposons (lower left panel), and DNA transposons (right panel). Each point corresponds to a copy. Copies with a divergence close to 0 and ratio close to 1 correspond to potentially active and full-length copies. As the divergence increases and ratio decreases, corresponding copies are more degraded. LINE, long interspersed nuclear element; LTR, long terminal repeat.

Another example of what can be directly performed using the outfiles *.transposons.csv and *.ltr.csv is displayed in Figure 5. The divergence of sequences (%_Div, column 2) was plotted against the size ratio for each copy compared with the reference element (%_of_Ref, column 17) for each superfamily in the euchromatin portion of the genome (chromosomes 2L, 2R, 3L, 3R, 4, and X). This procedure can allow the quality of the copies inserted into the genome to be determined quickly; that is*,* for a given family or superfamily, if the copies are mostly full-length (ratio close to 1) and not divergent from the reference, this could indicate recent insertion events. For example, in Figure 5, the elements from the *LTR/Copia* superfamily (including the families *copia*, *copia2*, *FROGGER*, and *1731*) mainly correspond to highly conserved copies (with a small divergence compared to their reference) with two populations of copies: one corresponding to almost full-length copies (potentially recent insertions) and the other corresponding to short copies. When looking in more detail, the populations of conserved copies of small sizes correspond mainly to *copia2* copies but do not represent solo-LTRs (see Additional file 5: Figure S1 for individual representation of *copia, copia2*, *FROGGER*, *and 1731* families). The same information can be produced for the other LTR-retrotransposon classes (Additional file 6: Figure S2 and Additional file 7: Figure S3 for individual family representations of *Gypsy* and *BEL/Pao* elements, respectively). Elements from the *LINE/LOA* superfamily, which in this case correspond to only one family (the *Baggins* family), had copies with low divergence compared to the reference but with different sizes, and a few of them were full-length, which could illustrate the same date of activity for the different copies and the transposition mechanism for LINE-like elements, which can be truncated at their 5′ end upon insertion. Thus, globally, we can easily obtain information concerning the population of copies of a given family and their positions in the genome.

### Test of the tool using the *H. sapiens* genome RepeatMasker output files

We retrieved the RepeatMasker .*out* file from the RepeatMasker website (http://www.repeatmasker.org), which was produced using the hg19 version (February 2009) of the genome sequence with the open-3.3.8 version of RepeatMasker and Repeat Library 20120124. This file contains results for all chromosomes, that is, 22 autosomal chromosomes and the two sex chromosomes (X and Y) that we considered in the test. We did not take into account results corresponding to randomly placed sequences, unplaced sequences (chrUn), and particular regions of chromosome 6 (corresponding to different haplotypes of the major histocompatibility complex region), chromosome 4, and chromosome 17.

### Determining the number of ambiguous cases that may require manual inspection (option --*dry-run*/--*choice*)

We determined the number of ambiguous cases that could be manually expertized for our file. For all of the considered chromosomes, a total of 12,133 possible choices appeared, which could potentially be investigated (see Additional file 8: Table S5 for the number by chromosomes). This large number indicates that complete manual annotation would be impossible to manage; however, by reducing the analysis to some TE families of interest, it would still be possible.

### Running the program with and without the --*strict* option

We ran our program with and without the --*strict* option. Table 2 displays the percent coverage for each TE class in each chromosome and the two cases. The average coverage for each TE class without the --*strict* option was congruent with the admitted TE content in the human genome with 3.23% DNA transposons, 19.85% LINEs, 13.16% SINEs, and 8.73% LTR-retrotransposons, representing a total of 44.98% TEs in the genome [1].

**Table 2:** **Percent coverage of each chromosome and for each class of TE in *H. sapiens *** **with and without the --*strict *** **option**

**Chromosome**	**Without --*strict *** **option**					**With --*strict *** **option**				
	**DNA**	**LINE**	**SINE**	**LTR**	**Total**	**DNA**	**LINE**	**SINE**	**LTR**	**Total**
Chr 1	3.28	20.03	14.30	8.35	45.96	1.48	8.75	10.48	4.73	25.44
Chr 2	3.86	22.56	11.89	9.27	47.58	1.62	10.19	8.83	5.06	25.70
Chr 3	4.05	23.50	12.14	9.95	49.64	1.68	11.60	8.63	5.44	27.35
Chr 4	3.72	24.45	10.26	11.61	50.04	1.67	12.40	7.30	6.49	27.86
Chr 5	3.86	23.86	11.34	10.1	49.16	1.61	11.91	8.13	5.46	27.11
Chr 6	3.82	23.17	11.57	9.72	48.28	1.68	11.23	8.74	5.66	27.31
Chr 7	3.55	21.73	13.88	9.00	48.16	1.54	9.80	11.13	5.20	27.67
Chr 8	3.63	22.93	11.98	10.12	48.66	1.56	10.65	8.59	5.57	26.37
Chr 9	3.10	19.50	12.18	7.90	42.68	1.28	8.94	8.92	4.29	23.43
Chr 10	3.67	20.79	13.91	8.50	46.87	1.52	9.20	10.59	4.82	26.13
Chr 11	3.42	22.95	13.47	9.10	48.94	1.45	10.85	9.12	5.13	26.55
Chr 12	3.75	21.62	14.91	9.55	49.83	1.53	10.03	11.27	5.31	28.14
Chr 13	3.08	19.23	8.53	8.95	39.79	1.35	8.62	6.41	4.99	21.37
Chr 14	3.03	18.53	11.4	8.24	41.20	1.35	8.79	8.65	4.70	23.49
Chr 15	3.02	17.61	12.52	6.30	39.45	1.26	7.42	9.65	3.28	21.61
Chr 16	3.12	14.55	18.05	7.32	43.04	1.03	5.52	14.18	3.58	24.31
Chr 17	3.20	15.36	21.55	6.48	46.59	1.33	5.45	17.97	3.53	28.28
Chr 18	3.62	21.54	10.74	9.21	45.11	1.52	9.08	8.00	4.97	23.57
Chr 19	2.13	13.43	26.98	8.58	51.12	0.74	5.09	24.11	5.27	35.21
Chr 20	4.17	18.84	15.90	8.70	47.61	1.50	7.15	11.79	3.71	24.15
Chr 21	2.50	14.53	8.30	8.97	34.30	1.01	6.18	6.67	5.01	18.87
Chr 22	1.95	10.95	15.33	4.64	32.87	0.79	3.34	12.02	2.39	18.54
Chr X	3.26	33.71	10.42	11.38	58.77	1.50	20.70	7.80	7.41	37.41
Chr Y	0.79	11.16	4.38	7.51	23.84	0.29	6.96	3.41	5.09	15.75
Average	3.23	19.85	13.16	8.73	44.98	1.34	9.16	10.10	4.88	25.48

LINE, long interspersed nuclear element; LTR, long terminal repeat; SINE, short interspersed nuclear element; TE, transposable element.

One original feature of our program is the ability to compute detailed quantitative information chromosome by chromosome, which differs from the output table produced by RepeatMasker. This feature allows us to show that the representation of each TE class differs according to the chromosome. For DNA transposons, chromosomes 3 and 20 displayed the highest proportion of these elements (4.05% and 4.17%, respectively), whereas the Y chromosome is particularly poor in elements of this class with only 0.79%. The X chromosome contains the highest proportion of LINEs and LTR-retrotransposons (33.71% and 11.38%, respectively) with chromosome 22 harboring the lowest proportion of the same elements (10.95% LINEs and 4.64% LTR-retrotransposons). Finally, SINEs are particularly abundant on chromosome 19 (26.98%) and rare on the Y chromosome (4.38%). Globally, the X chromosome has the highest proportion of TEs (58.77%), whereas the Y chromosome has the lowest proportion of TEs (23.84%). This observation is congruent with the discrepancy observed for particular families between the autosomal and sex chromosomes [29].

**Figure 6:**
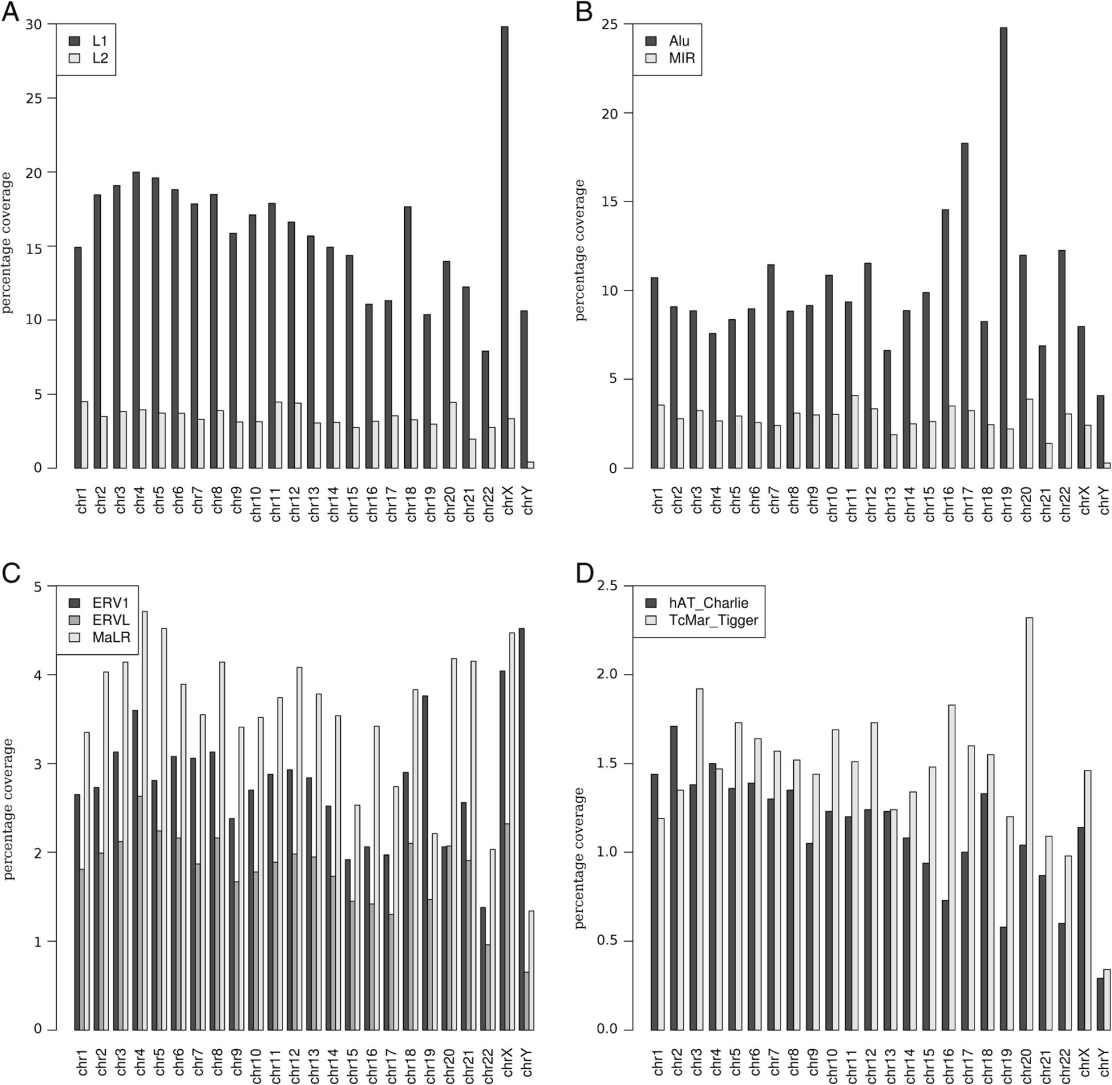
**Percent coverage of the most represented TE families in the human genome for each chromosome. (A)***L1* and *L2* elements are the two most represented LINE superfamilies with *L1* representing the highest proportion. **(B)***Alu* and *MIR* elements are the two most represented SINE superfamilies with the *Alu* representing the highest proportion. **(C)***ERV1*, *ERVL*, and *MaLR* are the three most represented LTR-retrotransposon superfamilies. **(D)***hAT_Charlie* and *TcMar_Tigger* are the most abundant DNA transposon superfamilies. LINE, long interspersed nuclear element; LTR, long terminal repeat; SINE, short interspersed nuclear element; TE, transposable element.

We examined the base coverage proportion for the most represented TE families in each chromosome (Figure 6). For each chromosome, the most represented LINEs mainly correspond to *L1* and then *L2* (Figure 6A). The two most represented SINE families include *Alu* and *MIR* (Figure 6B). Among the LTR-retrotransposons, the most represented elements correspond to the *MaLR* families in all chromosomes except chromosomes 19 and Y in which they correspond to the *ERV1* families. The *ERVL* families correspond to the third most represented LTR-retrotransposons in all chromosomes (Figure 6C). Among the DNA transposons, the *TcMar_Tigger* families are the most represented in all chromosomes with the exception of chromosomes 1 and 2 in which the *hAT_Charlie* families are the most abundant.

The same global distributions are observed when using the --*strict* option, which takes into account elements that follow our 80-80 rule. However, the global amount of each class decreases with an average of 25.48% of the genome (Table 2). The elements following this rule are expected to be well-conserved, suggesting that these elements were potentially active until recently. Indeed, the most represented families correspond to those known to have had a recent activity (Table 3) such as LINE *L1* and SINE *Alu*[26]. Among *Alu* elements, the most represented families correspond to *AluJb*, *AluSz*, *AluY*, *AluSx1*, and *AluSx*, which usually represent more than the half of the total *Alu*s*.* However, the most represented LTR-retrotransposons correspond to the *ERV1* and *MaLR* families, and only *ERVK* elements are supposed to remain active [30].

**Table 3:** **Percent coverage of each chromosome of the most represented TE families using the --*strict *** **option**

**Chromosome**	***L1***	***Alu***	***ERV1***	***ERVL-MaLR***	**Total**
Chr 1	8.73	10.42	2.07	1.76	22.98
Chr 2	10.18	8.80	2.13	2.11	23.22
Chr 3	11.59	8.59	2.46	2.09	24.73
Chr 4	12.38	7.28	2.80	2.54	25.00
Chr 5	11.89	8.10	2.17	2.38	24.54
Chr 6	11.21	8.71	2.47	2.13	24.52
Chr 7	9.79	11.11	2.47	1.89	25.26
Chr 8	10.64	8.56	2.48	2.11	23.79
Chr 9	8.93	8.88	1.81	1.73	21.35
Chr 10	9.19	10.55	2.19	1.78	23.71
Chr 11	10.84	9.07	2.31	1.92	24.14
Chr 12	10.02	11.22	2.35	1.98	25.57
Chr 13	8.61	6.39	2.17	2.08	19.25
Chr 14	8.78	8.62	1.96	1.96	21.32
Chr 15	7.41	9.61	1.46	1.25	19.73
Chr 16	5.51	14.13	1.63	1.33	22.60
Chr 17	5.43	17.91	1.63	1.27	26.24
Chr 18	9.07	7.97	2.18	2.01	21.23
Chr 19	5.08	24.07	2.90	0.87	32.92
Chr 20	7.13	11.71	1.61	1.49	21.94
Chr 21	6.17	6.65	1.98	2.23	17.03
Chr 22	3.33	11.98	1.06	0.83	17.20
Chr X	20.69	7.76	3.38	2.78	34.61
Chr Y	6.96	3.40	3.27	0.69	14.32

TE, transposable element.

## Conclusions

We have developed a tool to conveniently parse the classic RepeatMasker *.out* file to improve the original annotation provided, by including reconstruction of full-length copies. This information includes in particular a measure of the quality of the copies compared to a reference element, as well as the exact position and orientation of each copy and some quantification concerning their proportion in the genome/chromosome sequence, allowing for a fast and accurate assessment of the exact TE content. Additionally, the sequence of each copy with or without flanking sequences can be retrieved directly, allowing further analyses of the TEs. We hope that this tool will assist non-bioinformatics scientists in the more accurate identification of TE copies.

## Availability and requirements

Project name: One code to find them all.

Project home: http://doua.prabi.fr/software/one-code-to-find-them-all.

Operating system(s): Linux/Unix, Mac OS X, Windows (with Perl installed).

Programming language: Perl.

License: GNU General Public License.

## Abbreviations

LINE: Long interspersed nuclear element; LTR: Long terminal repeat; NGS: Next generation sequencing; SINE: Short interspersed nuclear element; TE: Transposable element; TSD: Target site duplication.

## Competing interests

The authors declare that they have no competing interests.

## Authors’ contributions

EL conceived the project; MBB implemented the programs; and EL, MBB, and AH tested the programs and wrote the manuscript. All authors read and approved the final manuscript.

## Supplementary Material

Additional file 1: Table S1 Number of ambiguous cases by chromosome for *D. melanogaster* (UCSC file). Table containing the number of ambiguous cases obtained for each chromosome for *D. melanogaster* using the RepeatMasker output file provided by the UCSC website. Click here for file

Additional file 2: Table S2 Copy number per chromosome for each category of TEs in *D. melanogaster* without the --*strict* option for *D. melanogaster* (UCSC file). Table containing the copy number per chromosome for each category of TEs in *D. melanogaster* without the *--strict* option for *D. melanogaster* using the RepeatMasker output file provided by the UCSC website. TE, transposable element. Click here for file

Additional file 3: Table S3 Percent coverage for all TEs on each chromosome of *D. melanogaster* without the --*strict* option. Table containing the percent coverage for all TEs on each chromosome of *D. melanogaster* without the *--strict* option for the two RepeatMasker output files (from UCSC and RepeatMasker websites). TE, transposable element. Click here for file

Additional file 4: Table S4 Percent coverage for all TEs on each chromosome of *D. melanogaster* (RepeatMasker version). Table containing the percent coverage for all TE classes on each chromosome of *D. melanogaster* using the RepeatMasker output file provided by the RepeatMasker website, with and without the *--strict* option. TE, transposable element. Click here for file

Additional file 5: Figure S1 Plot of the divergences according to the size ratio of elements from the *Copia* subfamily in *D. melanogaster*. Figure representing the divergence (column %_Div in file *.ltr.csv) of sequences plotted against the size ratio of the copy compared to the reference element (column %_of_Ref in file *.ltr.csv). Each point corresponds to a copy. Copies with a divergence close to 0 and ratio close to 1 correspond to potentially active and full-length copies. As the divergence increases and ratio decreases, corresponding copies are more degraded. Click here for file

Additional file 6: Figure S2 Plot of the divergences according to the size ratio of elements from the *Gypsy* subfamily in *D. melanogaster*. Figure representing the divergence (column %_Div in file *.ltr.csv) of sequences plotted against the size ratio of the copy compared to the reference element (column %_of_Ref in file *.ltr.csv). Each point corresponds to a copy. Copies with a divergence close to 0 and ratio close to 1 correspond to potentially active and full-length copies. As the divergence increases and ratio decreases, corresponding copies are more degraded. Click here for file

Additional file 7: Figure S3 Plot of the divergences according to the size ratio of elements from the *BEL/Pao* subfamily in *D. melanogaster*. Figure representing the divergence (column %_Div in file *.ltr.csv) of sequences has been plotted against the size ratio of the copy compared to the reference element (column %_of_Ref in file *.ltr.csv). Each point corresponds to a copy. Copies with a divergence close to 0 and ratio close to 1 correspond to potentially active and full-length copies. As the divergence increases and ratio decreases, corresponding copies are more degraded. Click here for file

Additional file 8: Table S5 Number of ambiguous cases by chromosome for *H. sapiens.* Table containing the number of ambiguous cases by chromosome for *H. sapiens.* Click here for file
