# Supplementary material for: “One code to find them all”: a perl tool to conveniently parse RepeatMasker output files
Source: Mob DNA. 2014 May 1;5:13. doi: 10.1186/1759-8753-5-13 (PMC4021974; doi:10.1186/1759-8753-5-13)
Supplement: Additional file 1: Table S1 — Number of ambiguous cases by chromosome for D. melanogaster (UCSC file). Table containing the number of ambiguous cases obtained for each chromosome for D. melanogaster using the RepeatMasker output file provided by the UCSC website. [file 1759-8753-5-13-S1.pdf]

Additional file 1: number of ambiguous cases by chromosome for *D. melanogaster* (UCSC file)

2L	30
2LHet	11
2R	52
2RHet	99
3L	46
3LHet	59
3R	8
3RHet	52
4	11
U	170
Uextra	301
X	14
XHet	6
YHet	3
**TOTAL**	**862**
