# Supplementary material for: “One code to find them all”: a perl tool to conveniently parse RepeatMasker output files
Source: Mob DNA. 2014 May 1;5:13. doi: 10.1186/1759-8753-5-13 (PMC4021974; doi:10.1186/1759-8753-5-13)
Supplement: Additional file 2: Table S2 — Copy number per chromosome for each category of TEs in D. melanogaster without the --strict option for D. melanogaster (UCSC file). Table containing the copy number per chromosome for each category of TEs in D. melanogaster without the --strict option for D. melanogaster using the RepeatMasker output file provided by the UCSC website. TE, transposable element. [file 1759-8753-5-13-S2.pdf]

**Additional file 2:** copy number per chromosome for each category of TEs in *D. melanogaster* without the *strict* option

| | | UCSC | | | | RM | | | |
|---|---|---|---|---|---|---|---|---|---|
| | **chromosomes** | **DNA** | **Non-LTR** | **LTR** | **Total** | **DNA** | **Non-LTR** | **LTR** | **Total** |
| **Euchromatin** | 2L | 252 | 907 | 494 | **1653** | 914 | 263 | 498 | **1675** |
| | 2R | 268 | 1066 | 702 | **2036** | 1018 | 343 | 707 | **2068** |
| | 3L | 237 | 997 | 773 | **2007** | 892 | 369 | 771 | **2032** |
| | 3R | 132 | 517 | 455 | **1104** | 549 | 125 | 455 | **1129** |
| | 4 | 128 | 575 | 39 | **742** | 626 | 77 | 35 | **738** |
| | X | 168 | 654 | 588 | **1410** | 720 | 187 | 585 | **1492** |
| | **Total** | **1185** | **4716** | **3051** | **8952** | **4719** | **1364** | **3051** | **9134** |
| **Heterochromatin** | 2LHet | 27 | 51 | 90 | **168** | 46 | 23 | 88 | **157** |
| | 2RHet | 232 | 677 | 805 | **1714** | 462 | 441 | 811 | **1714** |
| | 3LHet | 193 | 615 | 709 | **1517** | 463 | 339 | 708 | **1510** |
| | 3RHet | 182 | 514 | 677 | **1373** | 363 | 319 | 683 | **1365** |
| | Xhet | 19 | 98 | 21 | **138** | 65 | 54 | 21 | **140** |
| | Yhet | 20 | 71 | 87 | **178** | 46 | 45 | 89 | **180** |
| | **Total** | **673** | **2026** | **2389** | **5088** | **1445** | **1221** | **2400** | **5066** |
| **Unplaced** | U | 593 | 1956 | 3303 | **5852** | 909 | 1589 | 3365 | **5863** |
| | Uextra | 1744 | 9044 | 11387 | **22175** | 2132 | 8253 | 11827 | **22212** |
| | **Total** | **2337** | **11000** | **14690** | **28027** | **3041** | **9842** | **15192** | **28075** |
| **Total** | | **4195** | **17742** | **20130** | **42067** | **9205** | **12427** | **20643** | **42275** |
| **Total (without U and Uextra)** | | **1858** | **6742** | **5440** | **14040** | **6164** | **2585** | **5451** | **14200** |
