# Supplementary material for: “One code to find them all”: a perl tool to conveniently parse RepeatMasker output files
Source: Mob DNA. 2014 May 1;5:13. doi: 10.1186/1759-8753-5-13 (PMC4021974; doi:10.1186/1759-8753-5-13)
Supplement: Additional file 3: Table S3 — Percent coverage for all TEs on each chromosome of D. melanogaster without the --strict option. Table containing the percent coverage for all TEs on each chromosome of D. melanogaster without the --strict option for the two RepeatMasker output files (from UCSC and RepeatMasker websites). TE, transposable element. [file 1759-8753-5-13-S3.pdf]

Additional file 3: percent coverage for all TEs on each chromosome of *D. melanogaster* without the *strict* option

	USCS	RM
**2L**	6.78	6.78
**2LHet**	66.04	65.87
**2R**	8.76	8.80
**2RHet**	63.27	63.44
**3L**	8.13	8.16
**3LHet**	70.69	71.05
**3R**	3.60	3.60
**3RHet**	70.84	71.2
**4**	30.19	30.52
**U**	54.43	54.67
**Uextra**	43.92	44.14
**X**	6.07	6.10
**Xhet**	57.06	56.99
**Yhet**	40.72	41.21
