# Supplementary material for: “One code to find them all”: a perl tool to conveniently parse RepeatMasker output files
Source: Mob DNA. 2014 May 1;5:13. doi: 10.1186/1759-8753-5-13 (PMC4021974; doi:10.1186/1759-8753-5-13)
Supplement: Additional file 4: Table S4 — Percent coverage for all TEs on each chromosome of D. melanogaster (RepeatMasker version). Table containing the percent coverage for all TE classes on each chromosome of D. melanogaster using the RepeatMasker output file provided by the RepeatMasker website, with and without the --strict option. TE, transposable element. [file 1759-8753-5-13-S4.pdf]

Additional file 4: percent coverage for all TEs on each chromosome of *D. melanogaster* (RM version)

		**without *strict* option**				**with *strict* option**			
		DNA	Non-LTR	LTR	Total coverage	DNA	Non-LTR	LTR	Total coverage
Euchromatin	2L	1.38	1.51	3.89	6.78	1.21	1.41	3.52	6.13
	2R	1.53	2.26	5.02	8.80	1.33	2.08	4.33	7.74
	3L	1.20	2.31	4.64	8.16	1.07	2.20	4.00	7.27
	3R	0.56	0.68	2.36	3.60	0.46	0.66	2.27	3.39
	4	15.7	10.06	4.77	30.52	13.82	9.13	4.15	27.09
	X	1.03	1.09	3.97	6.10	0.94	1.05	3.68	5.67
Heterochromatin	2LHet	6.96	14.19	44.73	65.87	6.76	14.01	37.54	58.31
	2RHet	6.56	19.33	37.54	63.44	6.22	18.54	27.61	52.37
	3LHet	8.09	20.69	42.26	71.05	7.65	19.5	30.95	58.1
	3RHet	7.32	22.00	41.88	71.2	6.89	21.3	31.79	59.99
	Xhet	9.71	33.84	13.44	56.99	8.52	30.09	11.82	50.43
	Yhet	5.59	12.08	23.54	41.21	5.42	11.9	18.69	36.01
Unplaced scaffolds	U	4.41	16.54	33.72	54.67	4.28	15.81	26.47	46.55
	Uextra	2.75	17.97	23.43	44.14	2.68	17.45	20.58	40.71
