# Supplementary material for: “One code to find them all”: a perl tool to conveniently parse RepeatMasker output files
Source: Mob DNA. 2014 May 1;5:13. doi: 10.1186/1759-8753-5-13 (PMC4021974; doi:10.1186/1759-8753-5-13)
Supplement: Additional file 7: Figure S3 — Plot of the divergences according to the size ratio of elements from the BEL/Pao subfamily in D. melanogaster. Figure representing the divergence (column %_Div in file *.ltr.csv) of sequences has been plotted against the size ratio of the copy compared to the reference element (column %_of_Ref in file *.ltr.csv). Each point corresponds to a copy. Copies with a divergence close to 0 and ratio close to 1 correspond to potentially active and full-length copies. As the divergence increases and ratio decreases, corresponding copies are more degraded. [file 1759-8753-5-13-S7.pdf]

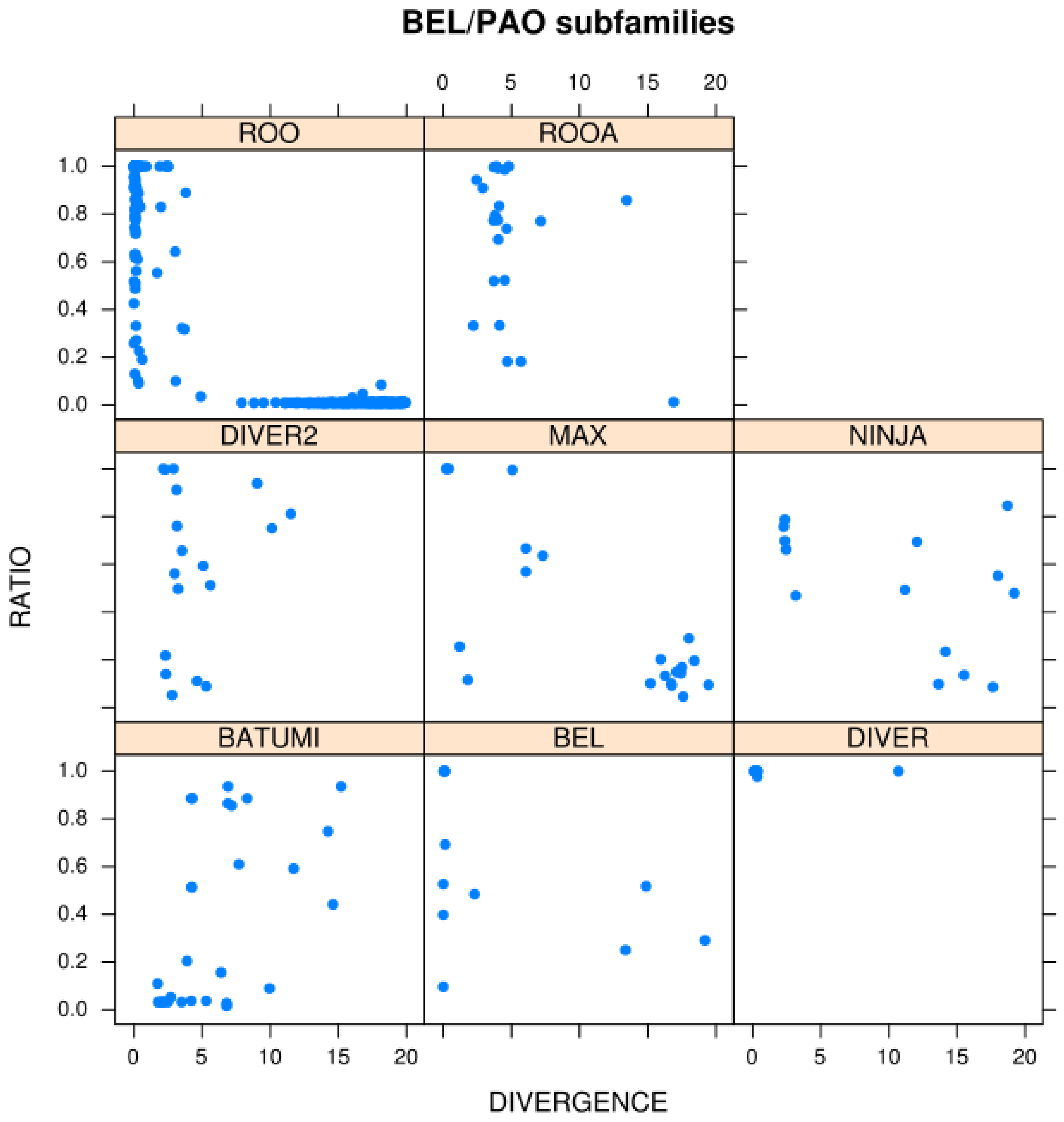

Additional file 7: Plot of the divergences according to the size ratio of elements from the *BEL/Pao* subfamily. The divergence (column %_Div in files **.ltr.csv*) of sequences has been plotted against the size ratio of the copy compared to the reference element (column %_of_Ref in files **.ltr.csv*). Each point corresponds to a copy. Copies with a divergence close to 0 and ratio close to 1 correspond to potentially active and full-length copies. As the divergence increases and ratio decreases, corresponding copies are more degraded.
