# Supplementary material for: “One code to find them all”: a perl tool to conveniently parse RepeatMasker output files
Source: Mob DNA. 2014 May 1;5:13. doi: 10.1186/1759-8753-5-13 (PMC4021974; doi:10.1186/1759-8753-5-13)
Supplement: Additional file 8: Table S5 — Number of ambiguous cases by chromosome for H. sapiens. Table containing the number of ambiguous cases by chromosome for H. sapiens. [file 1759-8753-5-13-S8.pdf]

Additional file 8: number of ambiguous cases by chromosome for *H. sapiens*

chr1	1067
chr2	890
chr3	782
chr4	781
chr5	725
chr6	641
chr7	631
chr8	599
chr9	549
chr10	546
chr11	588
chr12	569
chr13	359
chr14	376
chr15	344
chr16	345
chr17	335
chr18	271
chr19	253
chr20	316
chr21	124
chr22	169
chrX	750
chrY	123
**TOTAL**	**12133**
